# Caveolin-1 is dispensable for early lymphoid development, but plays a role in the maintenance of the mature splenic microenvironment

**DOI:** 10.1186/s13104-018-3583-3

**Published:** 2018-07-13

**Authors:** Tyler A. Herek, Jacob E. Robinson, Tayla B. Heavican, Catalina Amador, Javeed Iqbal, Christine E. Cutucache

**Affiliations:** 1 Eppley Institute, University of Nebraska Medical Center, Omaha, NE USA; 2 Department of Biology, University of Nebraska at Omaha, 6001 Dodge St, Omaha, NE 68182 USA; 3 Department of Pathology and Microbiology, University of Nebraska Medical Center, Omaha, NE USA

**Keywords:** Caveolin-1, Immunophenotype, Spleen, B cell

## Abstract

**Objective:**

Caveolin-1 (CAV1) is known for its role as both a tumor suppressor and an oncogene, harboring a highly context-dependent role within a myriad of malignancies and cell types. In an immunological context, dysregulation of CAV1 expression has been shown to alter immunological signaling functions and suggests a pivotal role for CAV1 in the facilitation of proper immune responses. Nonetheless, it is still unknown how *Cav1*-deficiency and heterozygosity would impact the development and composition of lymphoid organs in mice. Herein, we investigated the impacts of *Cav1*-dysregulation on the lymphoid organs in young (12 weeks) and aged (36 weeks) *Cav1*^+*/*+^, *Cav1*^+*/*−^, and *Cav1*^−*/*−^ mice.

**Results:**

We observed that only *Cav1*-deficiency is associated with persistent splenomegaly at all timepoints. Furthermore, no differences in overall body weight were detected (and without sexual dimorphisms). Both aged *Cav1*^+*/*−^ and *Cav1*^−/−^ mice present with decreased CD19^+^CD22^+^ B cells and secondary-follicle atrophy, specifically in the spleen, compared with wild-type controls and irrespective of splenomegaly status. Consequently, the demonstrated effects on B cell homeostasis and secondary follicle characteristics prompted our investigation into follicle-derived human B-cell lymphomas. Our investigation points toward CAV1 as a dysregulated protein in follicle-derived B-cell malignancies without harboring a differential expression between more aggressive and indolent hematological malignancies.

**Electronic supplementary material:**

The online version of this article (10.1186/s13104-018-3583-3) contains supplementary material, which is available to authorized users.

## Introduction

Caveolin-1 (CAV1) is located on chromosome 7q31.2 at the D7S522 locus, a fragile point known to be deleted in certain human cancers [1]. *CAV1* is commonly described with a two-faced nature as both a tumor suppressor and an oncogene [2]. This paradox is seen across multiple malignancies as the expression of *CAV1* has been shown to be both up- and down-regulated compared to normal tissue, with both phenotypes capable of harboring poor prognoses [2, 3].

With the “Janus-faced” nature of *CAV1*, researchers have turned to *Cav1*-deficient mice to sort out the heterogeneity. *Cav1*^−*/*−^ mice, but not *Cav1*^+*/*−^ mice, harbor a reduced lifespan in comparison to wild-type (WT) controls; however, the reduction in lifespan is not due to an increased frequency of tumorigenic events [4, 5]. Rather, pulmonary and cardiac complications drive the observed lifespan reduction [4]. It is clear that *Cav1* plays a largely context-dependent role, with varied phenotypes seen throughout different organ systems as well as a progression of these phenotypes over time. Nonetheless, it is still unknown how *Cav1*-deficiency and heterozygosity would impact the development and composition of the primary and secondary lymphoid organs of mice. *Cav1* is expressed in cells of the immune system [6, 7] and plays a vital role in not only immune synapse formation [8] but also in macrophage-, T-, and B-cell signaling [8–14]. While previous studies have investigated *Cav1*-deficiency in terms of B-cell development [10, 14, 15], T-cell development [10, 11], and recently the immunophenotype of the spleen (SP) and bone marrow (BM) in aged mice [14]; no comprehensive study which includes both primary and secondary lymphoid organs, a longitudinal component, and a *Cav1*-heterozygous experimental population exists.

Consequently, herein we investigated the impact of *Cav1* on the lymphoid organ system. Specifically, we addressed how the primary and secondary lymphoid organs of mice can be immunophenotypically described at a young/non-diseased stage (12 weeks) and how the underlying pathologies of *Cav1*-deficiency impact these organs at near end-of-life (36 weeks).

## Main text

### Results

#### *Caveolin*-*1*-deficiency is associated with persistent splenomegaly

**Fig. 1:**
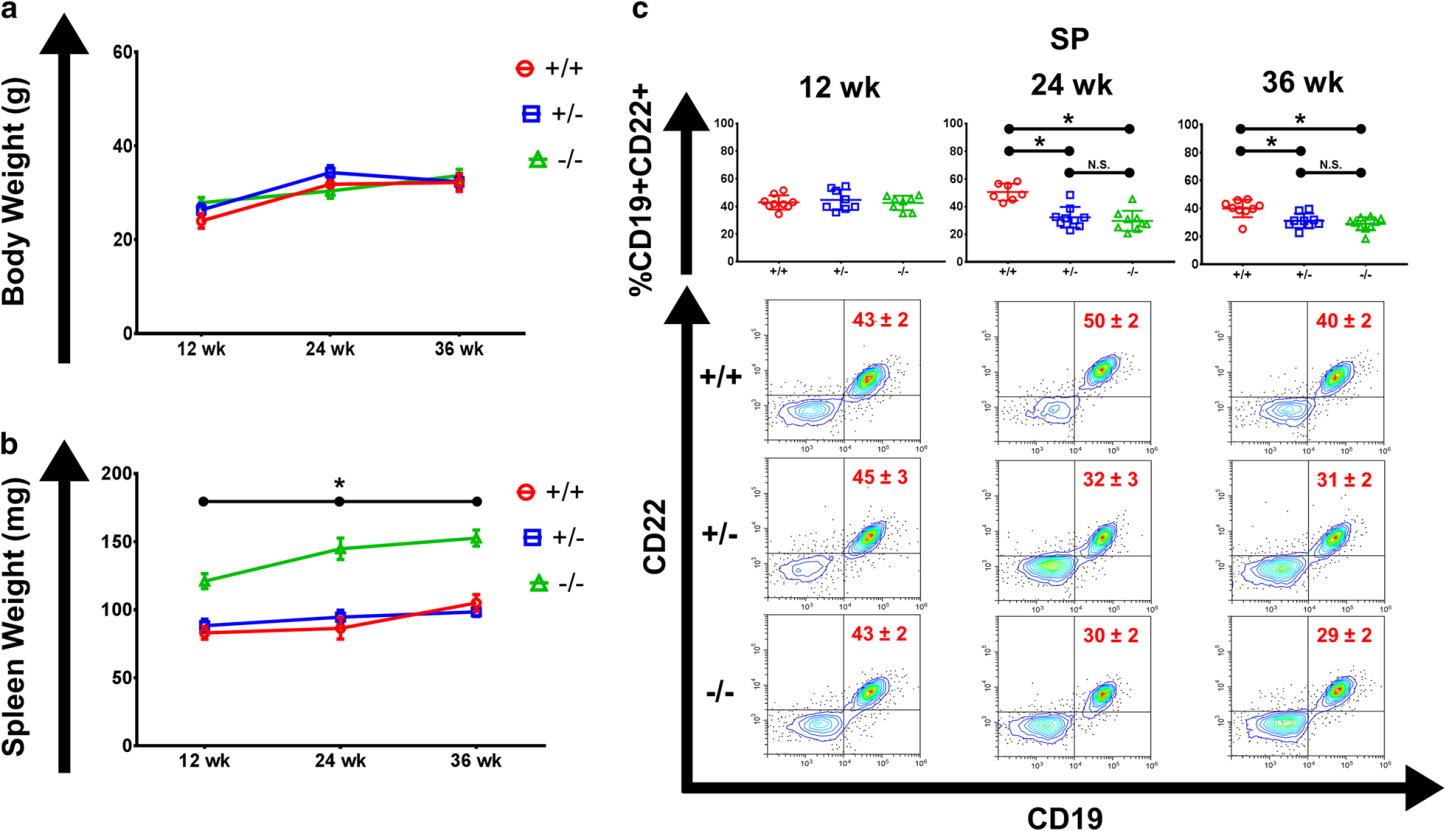
*Caveolin*-1 deficiency is associated with splenomegaly with implications for *Caveolin*-1 as haploinsufficient for splenic B-cell maintenance. **a** Line graph of mean body weight (g) of *Cav1*^+*/*+^ (red), *Cav1*^+*/*−^ (blue), and *Cav1*^−*/*−^ (green) mice at indicated timepoint (n = 9–22 per group). **b** Line graph of mean SP weight (mg) of *Cav1*^+*/*+^ (red), *Cav1*^+*/*−^ (blue), and *Cav1*^−*/*−^ (green) mice at indicated timepoint (n = 9–15 per group). **c** (Top panel) Dot plots for CD19^+^CD22^+^ B cells in the SP for listed genotypes at 12, 24, and 36 weeks (n = 7–10 per group). (Bottom panel) Representative contour flow cytometry plots for CD19^+^CD22^+^ B cells at listed genotypes for listed timepoints. (Line graphs show mean ± SEM, dot plots show mean ± SD with each dot corresponding to a biological replicate, flow cytometry gate values show mean ± SEM, *NS* not significant, *p < 0.05 ANOVA and Tukey post hoc test)

We observed no statistically significant differences in body weight (g) across genotypes and timepoints, with no sexual dimorphism (Fig. 1a, Additional file 1: Figure S1a). However, as early as 12 weeks, *Cav1*^−*/*−^ mice presented with splenomegaly; a finding consistent across all measured timepoints in comparison to WT mice, with *Cav1*^+*/*−^ mice mirroring WT mice and no observed sexual dimorphism (Fig. 1b, Additional file 1: Figure S1b).

#### *Caveolin*-*1* is dispensable for early lymphoid development but critical for B cell maintenance in the spleen

Investigating the impact of *Cav1*-deficiency and heterozygosity on B- and T-cell development and homeostasis, we observed no statistically significant differences in the pre/pro B-cell populations, immature B-cell populations, or overall CD19^+^ B-cell populations in the BM nor CD4/CD8 thymic populations in all experimental groups and time points (Additional file 2: Figure S2a, b). There were no observed changes in the resident CD3^+^ T-cell populations or skewing of the CD3^+^CD4^+^ versus CD3^+^CD8^+^ T-cell populations in the thymus (Thy), SP, lymph node (LN), or BM (Additional file 3: Figure S3a, b). Further, we observed no differences in the percentages of mature B cells in the LN or BM at 12 or 36 weeks (Additional file 3: Figure S3c).

Conversely, our aged *Cav1*^−*/*−^ and *Cav1*^+*/*−^ mice exhibited decreased CD19^+^CD22^+^ B-cell percentages in the SP as early as 24 weeks with corroborative results at 36 weeks (Fig. 1c). Our observation of a decrease in splenic B-cell percentages was seen without any concurrent changes to additionally analyzed white pulp populations, including: T cells (Additional file 3: Figure S3) or myeloid cells (Additional file 4: Figure S4a–c). With our flow cytometry controlled for cell input, events/sample (see “Methods”), and no change observed in overall lymphoid percentage (Additional file 4: Figure S4d), this excludes the splenomegaly of the *Cav1*-deficient mice as a confounding factor.

#### Aged *Cav1*-transgenic mice exhibit an altered splenic microenvironment

**Fig. 2:**
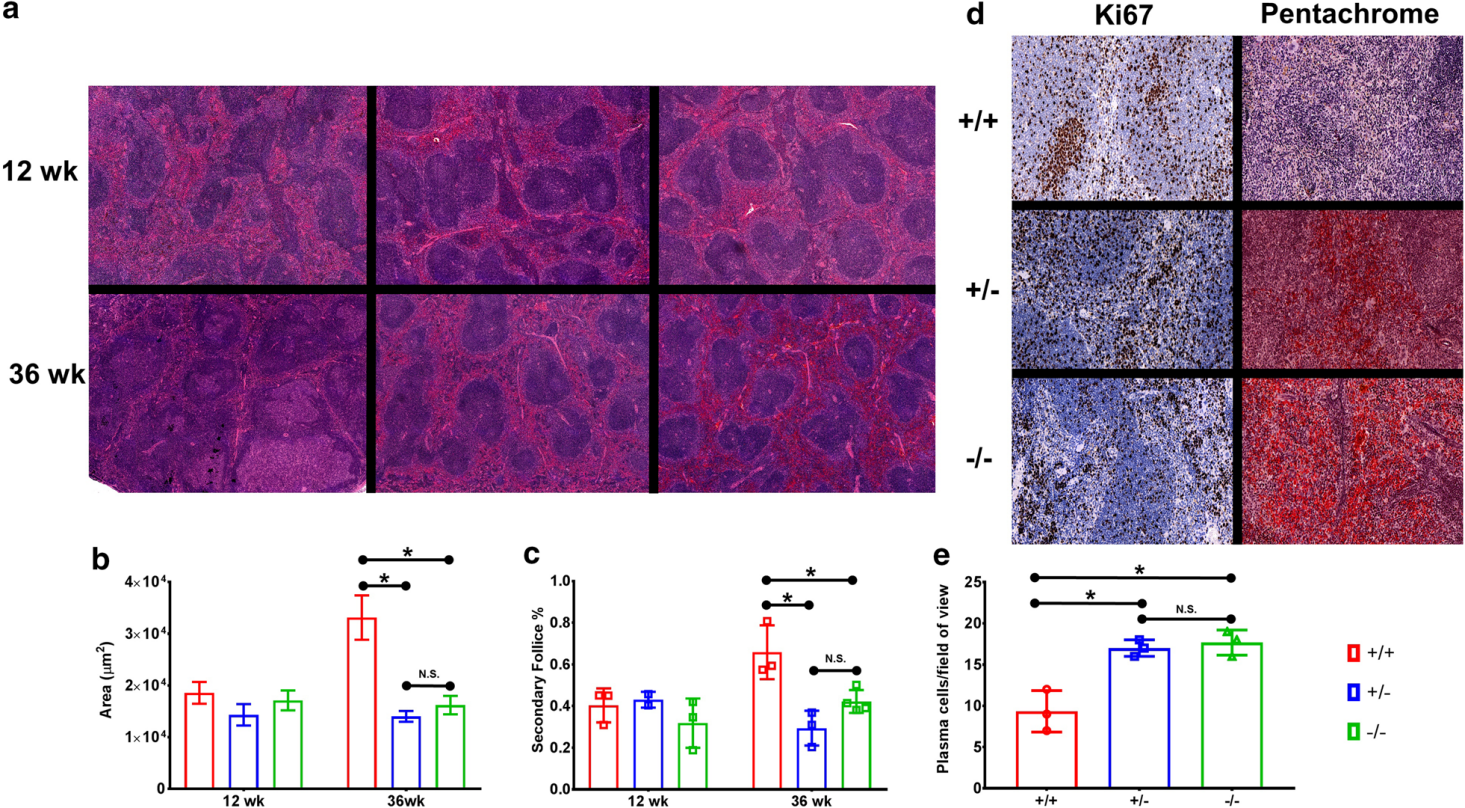
Aged Cav1-transgenic mice exhibit an altered splenic microenvironment. **a** Representative H&E stained sections of *Cav1*^+*/*+^, *Cav1*^+*/*−^, and *Cav1*^−*/*−^ spleens at ×4 magnification. **b** Bar graph of the mean splenic secondary follicle area (μm^2^) for listed genotypes at 12 and 36 weeks (n = 5–10 follicles across n = 2–4 mice per group). **c** Percentage of observable secondary follicles for listed genotypes at 12 and 36 weeks (n = 2–4 per group). **d** Representative Ki67 and Movat’s Pentachrome stained histological sections from 36-week spleens for listed genotypes at 20× magnification (n = 3 per group). Increased fibrin staining is visualized in bright red. **e** Bar graph of mean plasma cells identified per field of view (×40) for listed genotypes from 36-week splenic sections (n = 3 per group). (Bar graphs show mean ± SD, with each dot corresponding to a biological replicate *NS* not significant, *p < 0.05 ANOVA and Tukey post hoc test)

In response to the observed dysregulation of splenic B-cells in both *Cav1*^−*/*−^ and *Cav1*^+*/*−^- mice, we conducted histopathologic analysis on splenic sections from all genotypes. Spleens from 12-week mice showed no differences under routine examination of H&E sections (Fig. 2a) with normal splenic architecture, including: similar frequency of observable secondary follicles (Fig. 2b) as well as mean secondary follicle area (Fig. 2c) in accordance with previous observations [10]. However, at 36 weeks, we noted *Cav1*^−*/*−^ and *Cav1*^+*/*−^ mice to present with fewer secondary follicles (Fig. 2a, b) with the observed follicles to be smaller in comparison to age-matched splenic sections from WT mice (Fig. 2c).

Additional investigation into the proliferative indexes of the 36-week sections revealed no statistically significant differences in Ki67^+^ cells per field of view (Fig. 2d). Utilizing pentachrome staining we noted an increase in fibrin deposits in the spleens of *Cav1*^+*/*−^ and *Cav1*^−*/*−^ mice compared to WT spleens (Fig. 2d). No other investigated organs were observed to harbor these deposits. Interestingly, further histopathologic analysis revealed an increase in inter-follicular plasma cells in the spleens of *Cav1*^+*/*−^ and *Cav1*^−*/*−^ mice compared to WT mice (Fig. 2e).

#### Follicle center B-cell lymphomas display altered CAV1 staining and expression patterns

**Fig. 3:**
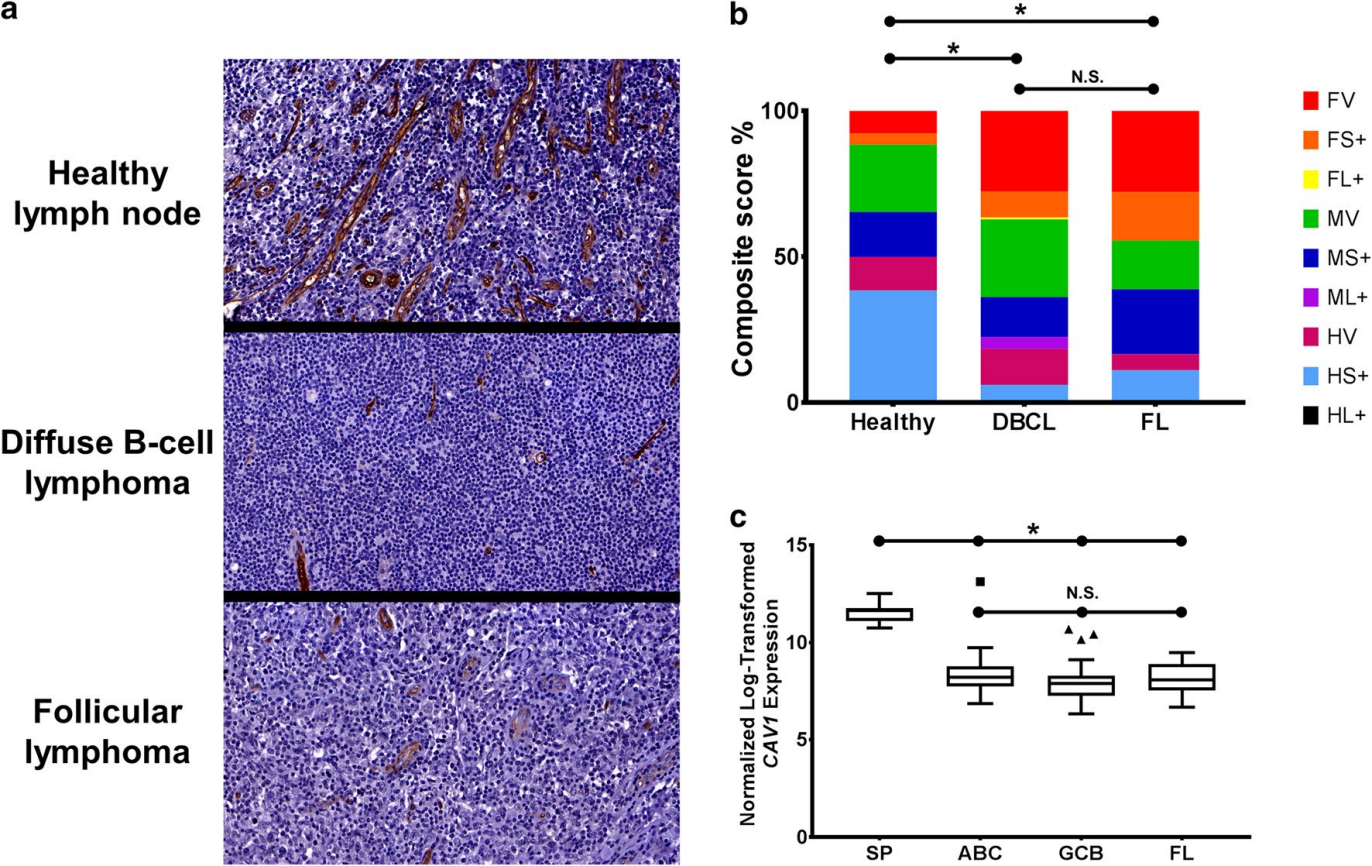
Follicle center B-cell lymphomas display altered CAV1 staining and expression patterns. **a** Representative tissue microarray cores stained for CAV1 expression. All images taken at ×20. **b** Bar graph of the distribution of composite scores assigned to healthy LN (n = 26), DBCL (n = 293), and FL (n = 18) samples for CAV1 staining. **c** Boxplot of the normalized log-transformed *CAV1* expression value using the 212097_at signal probe for SP (n = 12), diffuse large B-cell lymphoma activated B cell-like (DLBCL-ABC) (n = 46), diffuse large B-cell lymphoma germinal center B cell-like (DLBCL-GCB) (n = 40), and FL (n = 27). Line within box represents median, whiskers extend to Tukey lines. (For (**b**): *NS* not significant, please see “Methods” for description of scoring key, *p < 0.05 Kruskal–Wallis and Dunn’s multiple comparisons test. For (**c**): *NS* not significant, *p < 0.001 comparison among groups of arrays method)

Demonstrated above, we observed *Cav1* to play a role in the long-term homeostasis of the splenic B-cell population and splenic follicle morphology. We therefore became interested in investigating the expression of CAV1 in post-secondary follicle B-cell malignancies. To this end we scored the Biomax LY6161 tissue microarray for CAV1 staining intensity and localization comparing healthy lymph node samples to both diffuse B-cell lymphoma (DBCL) and follicular lymphoma (FL) samples (Fig. 3a). The distribution of staining scores for disease entities differed in comparison to the normal LNs with fewer B-cell malignancies scored for heavy stromal staining and an increase in those scored for faint blood/lymphatic vessel staining (Fig. 3b). There was no significant difference in the distribution of staining scores when comparing malignancies. Using publicly available microarray data we examined the expression of *CAV1* in tumor biopsy samples from FL and two established subtypes of diffuse large B-cell lymphoma (DLBCL), comparing them to normal SP controls. All malignancies analyzed had a lower mean expression of *CAV1* compared to the healthy splenic tissue (Fig. 3c).

### Discussion

Herein, we include a characterization of *Cav1* in the lymphoid compartments of a murine model. *Cav1*-deficient mice exhibited persistent splenomegaly compared to *Cav1*-heterozygous and WT mice. While the mechanism for *Cav1*-mediated splenomegaly is unknown, we ruled out a higher proliferative index as there were no differences found in Ki67^+^ staining numbers between genotypes. Previous investigations found very modest or no persistent differences in proliferation/apoptosis in *Cav1*-deficient spleens [5, 9, 14], suggesting a currently unresolved physiological condition may be causing the phenotype. However, a full knockout of *Cav1* is required to drive the splenomegaly as our data suggests that *Cav1*-heterozygosity is not sufficient to drive the dysregulation as splenomegaly was not observed in *Cav1*^+*/*−^ mice.

Previous investigations have established CAV1 as vital to T-cell function in the context of both antigen presentation and signal transduction [8, 11–13]. However, we and others demonstrate that *Cav1* is seemingly dispensable for T-cell development and homeostasis [10, 11, 13, 14].

We present data in support of a role for *Cav1* in the regulation of B-cell processes by observing a decrease in B-cell percentages, specifically in the SP, in aged *Cav1*-transgenic mice on the C57BL/6 background utilizing the knockout method described by Razani et al. [16]. This decrease was seen in conjunction with aberrant secondary follicle characteristics in aged mice, however a more detailed analysis of these changes is still needed. These findings are highlighted with our observation of a concurrent decrease in splenic B-cell percentages and an increase in inter-follicular plasma cells and fibrin deposits in *Cav1*-deficient and heterozygous mice without both exhibiting splenomegaly. This suggests that *Cav1*-heterozygosity is sufficient to drive the dysregulation of B-cell homeostasis and impair the Cav1-mediated internalization of fibrin [17], most likely caused by the angiogenic defects observed in *Cav1*-transgenic mice [16]. Further, the observation of increased inter-follicular plasma cells may elude to a basis for the decrease in CD19^+^CD22^+^ (i.e. mature, non-terminally differentiated) B cells in *Cav1*-transgenic mice without additionally perturbed populations as plasma cells (mature, terminally differentiated) downregulate pan-B surface markers during differentiation [18, 19]. Collectively, the decrease of mature B cells in conjunction with an increase of fibrin deposits and presence of plasma cells indicate that dysregulation of *Cav1* may induce an inflammatory splenic microenvironment.

While there is controversy precise role of *Cav1* in B-cell signaling, one point of agreement between all published reports is that there exists a role for *Cav1* in B-cell processes as evident by conserved dysregulated responses and changes to physiological populations over time [14, 15]. However, the lack of consistency in the results derived from established *Cav1*-knockout models calls into question how the method and selection of exon as well as the genetic background of the mouse contributes to the observed results.

Taken together, the data suggests that loss of *Cav1* alone is not sufficient to drive a robust immunological phenotype despite its frequent presence as a dysregulated molecule in human malignancies. However, *Cav1*-dysregulated cells appear “predisposed” to display aberrant signaling profiles given proper stimuli [10, 11, 13–15, 20–22]. This predisposition, or reprogramming, suggests *CAV1* as a facilitator of immune/inflammatory-related processes and could point toward its ultimate role in the progression of multiple human malignancies, including both solid tumor [23–25] and hematological diseases [8, 26, 27]. To this end, we examined the staining pattern of CAV1 and its expression in follicle-derived B-cell malignancies. We observed fainter, less stromal-localized CAV1 staining in the hematological malignancies with lower *CAV1* expression in tumor biopsies compared to normal splenic tissue. However, no differences were observed between the more aggressive DLBCL cases when compared to the more indolent FL cases, not forming a parallel between lowered CAV1 expression being associated with a more aggressive disease in both breast [23] and prostate cancer [24]. These results suggest that CAV1 is dysregulated in follicle-derived B cell malignancies but does not play an overt role between more aggressive and indolent disease entities.

### Methods

#### Flow cytometry

Leukocyte populations were isolated using Lympho Separation Medium (MP Biomedicals, USA) with red blood cells lysed using RBC lysis buffer (Alfa Aesar, USA). Cells were counted and checked for viability (> 95%) using Trypan Blue (STEMCELL Technologies, CA) and 10^6^ live cells were aliquoted for antibody staining (Additional file 1). Samples were run on a Cytoflex flow cytometer (Beckman Coulter, USA) and analyzed using the CytExpert 2.0 software (Beckman Coulter). Compensation was calculated utilizing a VersaComp antibody capture bead kit (Beckman Coulter). Cells were gated based off forward-side scatter, unstained controls, and fluorescence minus one controls. All samples are representative of at least 5 × 10^4^ events.

#### Histology

Animal care protocol found in Additional file 6: Additional methods. Harvested organs were placed into 10% neutral buffered formalin and allowed to fix for ≥ 72 h before preservation in 100% ethanol. Preserved tissues were paraffin-embedded and sectioned (4 μm). H&E and Movat’s pentachrome staining were conducted using standard procedures, Ki67 staining using an auto-staining system.

Histological scoring was conducted for Ki67 staining, secondary follicle counting, and plasma cell identification. In each instance, three independent researchers scored the relevant variable in 4 separate fields of view (n = 3 per genotype). All slides were viewed at identical magnifications. For scoring agreement metrics and follicle area quantification, please see Additional file 5: Table S1, Additional file 6: Additional methods.

#### TMA scoring

The LY6161 high-density lymphoma and normal lymph node tissue array was utilized as previously described [27]. Cases were scored for both intensity of stain (faint, moderate, heavy) and type of staining observed (vessel only, stromal + vessel, or lymphocyte + stromal + vessel). Patient characteristics and scoring agreement described in Additional file 5: Tables S1, S2.

#### Gene expression data

(.CEL) files were downloaded via NCBI Gene Expression Omnibus and uploaded into BRB-ArrayTools. Samples were collated and normalized using the MAS5.0 method. Samples described in Additional file 5: Table S3.

## Limitations

- Unresolved splenomegaly in *Cav1*^−*/*−^ mice.
- More in-depth germinal center investigation requires additional IHC markers.
- Higher resolution of B/T-populations could be achieved with additional flow markers.

## Additional files

**Additional file 1: Figure S1.** (**a**) Bar graph of mean body (g) of listed genotypes separated by males (no fill pattern) and females (fill pattern) (n = 3–9 per group). (**b**) Bar graph of mean SP weight (mg) of listed genotypes separated by males (no fill pattern) and females (fill pattern) (n = 2–11 per group). (Bar graphs show mean ± SD with each dot corresponding to a biological replicate, *NS* not significant, * = p < 0.05 ANOVA and Tukey post hoc test).

**Additional file 2: Figure S2.** (**a**) Dots plots of B-cell populations in the BM for *Cav1*^+*/*+^, *Cav1*^+*/*−^, and *Cav1*^−*/*−^ mice at 12 and 36 weeks (n = 8–13 per group). Pre/Pro B cells designated as CD19^+^CD22^−^, immature B cells designated as CD19^+^CD22^+^. (**b**) Representative pseudo-color flow cytometry plots of CD4^+^ and CD8^+^ populations in the Thy at 12 and 36 weeks for listed genotypes. (**c**) (Dot plots show mean ± SD with each dot corresponding to a biological replicate, flow cytometry gate values show mean ± SD).

**Additional file 3: Figure S3.** (**a**) Dot plots of CD3^+^ T cells in the Thy, BM, SP, and LN for listed genotypes at 12 and 36 weeks as determined by flow cytometry (n = 7–13 per group). (**b**) Dot plots of CD3^+^CD4^+^ and CD3^+^CD8^+^ T cell populations in the secondary lymphoid organs of listed genotype at 12 and 36 weeks (n = 4–9 per group). (**c**) Dot plots of CD19^+^CD22^+^ B cells in the LN for listed genotypes at 12 and 36 weeks (n = 8–11 per group). (Dot plots show mean ± SD with each dot corresponding to a biological replicate).

**Additional file 4: Figure S4.** (**a)** Dot plots of CD11b^+^ cells in the SP and BM at the listed genotypes for 12 and 36 weeks. (**b**) Dot plots of CD14^+^ cells in the SP and BM at the listed genotypes for 12 and 36 weeks. (**d**) Dot plot of splenic lymphocyte percentage in 36-week mice for listed genotypes. (Dot plots show mean ± SD with each dot corresponding to a biological replicate, *NS* not significant, * = p < 0.05 ANOVA and Tukey post hoc test).

**Additional file 5: Table S1.** Histological scoring table. **Table S2.** Patient characteristics from the LY6161 high-density lymphoma tissue array. **Table S3.** Publicly available GEO DataSets utilized for gene expression analysis. **Table S4.** Antibodies utilized within the study.

**Additional file 6.** Animal care and secondary follicle area determination methodologies are described.

## Abbreviations

CAV1: Caveolin-1
WT: wild-type
BM: bone marrow
SP: spleen
Thy: thymus
LN: lymph node
H&E: hematoxylin and eosin
DBCL: diffuse B-cell lymphoma
FL: follicular lymphoma
DLBCL: diffuse large B-cell lymphoma
DLBCL-ABC: diffuse large B-cell lymphoma activated B cell-like
DLBCL-GBC: diffuse large B-cell lymphoma germinal center B cell-like
N.S.: not significant
