# Supplementary figures and images for: Caveolin-1 is dispensable for early lymphoid development, but plays a role in the maintenance of the mature splenic microenvironment
Source: BMC Res Notes. 2018 Jul 13;11:470. doi: 10.1186/s13104-018-3583-3 (PMC6043983; doi:10.1186/s13104-018-3583-3)

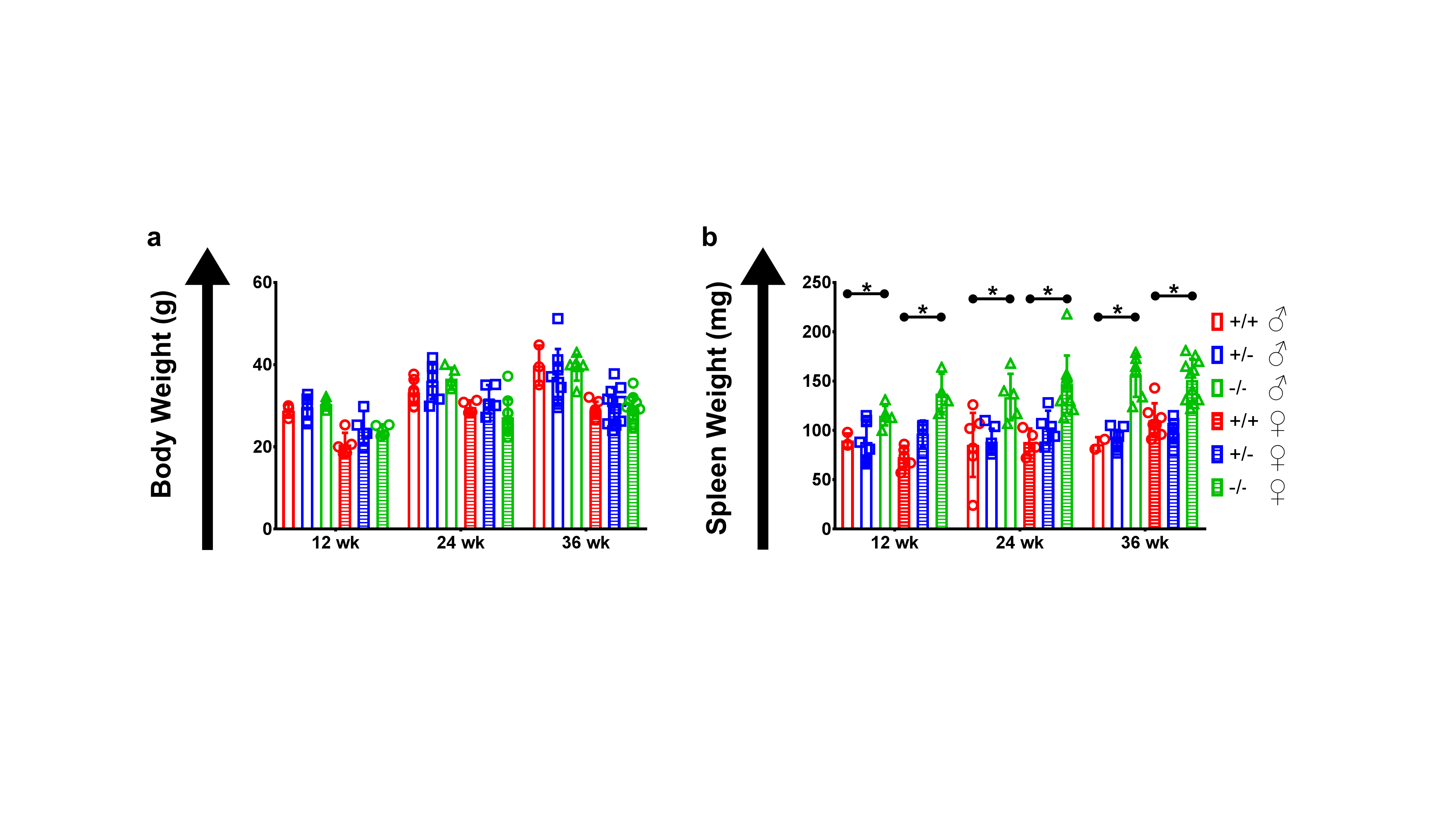

Supplement: Supplementary file 1 — Additional file 1: Figure S1. (a) Bar graph of mean body (g) of listed genotypes separated by males (no fill pattern) and females (fill pattern) (n = 3–9 per group). (b) Bar graph of mean SP weight (mg) of listed genotypes separated by males (no fill pattern) and females (fill pattern) (n = 2–11 per group). (Bar graphs show mean ± SD with each dot corresponding to a biological replicate, NS not significant, * = p < 0.05 ANOVA and Tukey post hoc test). [file 13104_2018_3583_MOESM1_ESM.tif]

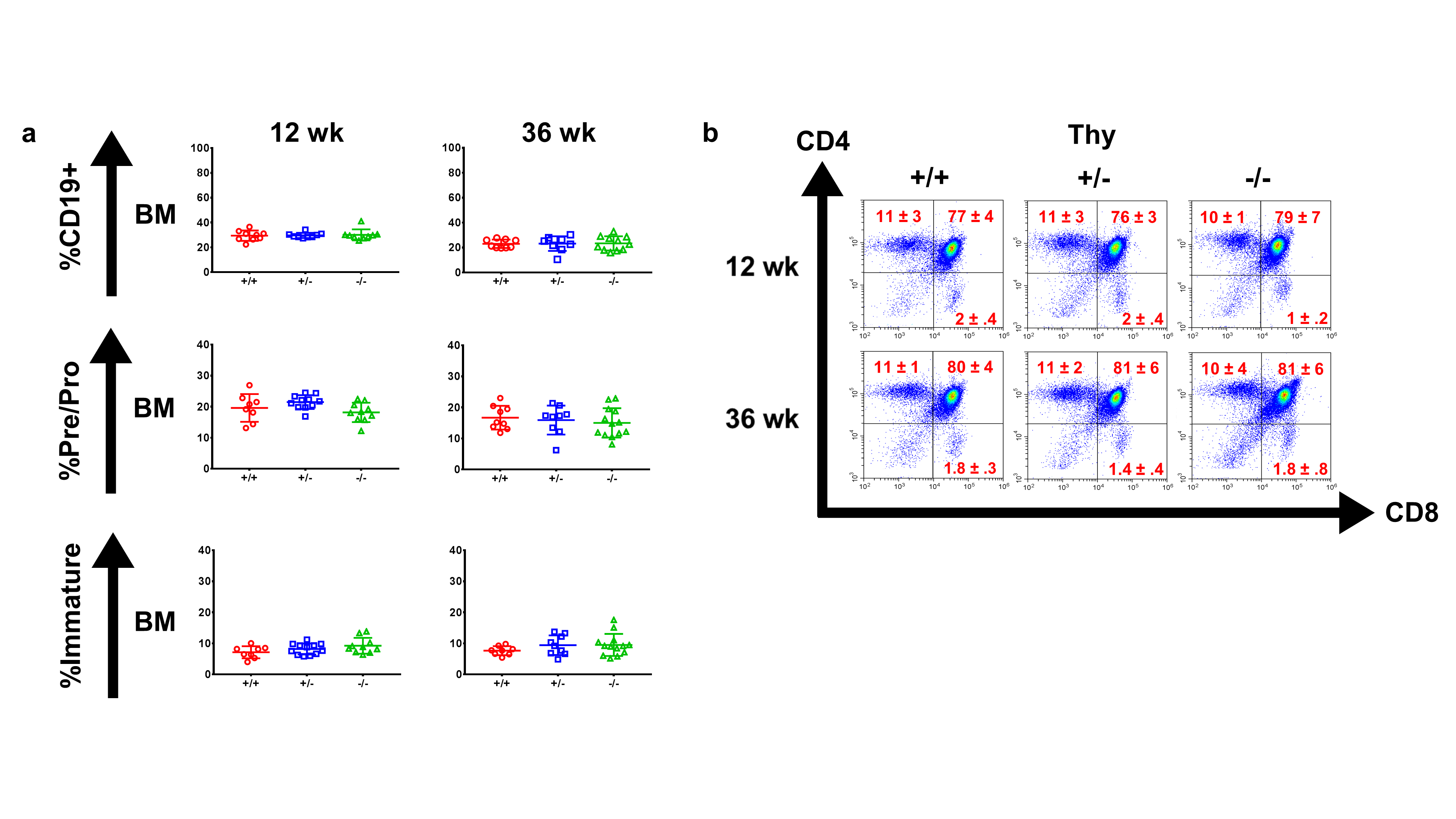

Supplement: Supplementary file 2 — Additional file 2: Figure S2. (a) Dots plots of B-cell populations in the BM for Cav1+/+, Cav1+/−, and Cav1−/− mice at 12 and 36 weeks (n = 8–13 per group). Pre/Pro B cells designated as CD19+CD22−, immature B cells designated as CD19+CD22+. (b) Representative pseudo-color flow cytometry plots of CD4+ and CD8+ populations in the Thy at 12 and 36 weeks for listed genotypes. (c) (Dot plots show mean ± SD with each dot corresponding to a biological replicate, flow cytometry gate values show mean ± SD). [file 13104_2018_3583_MOESM2_ESM.tif]

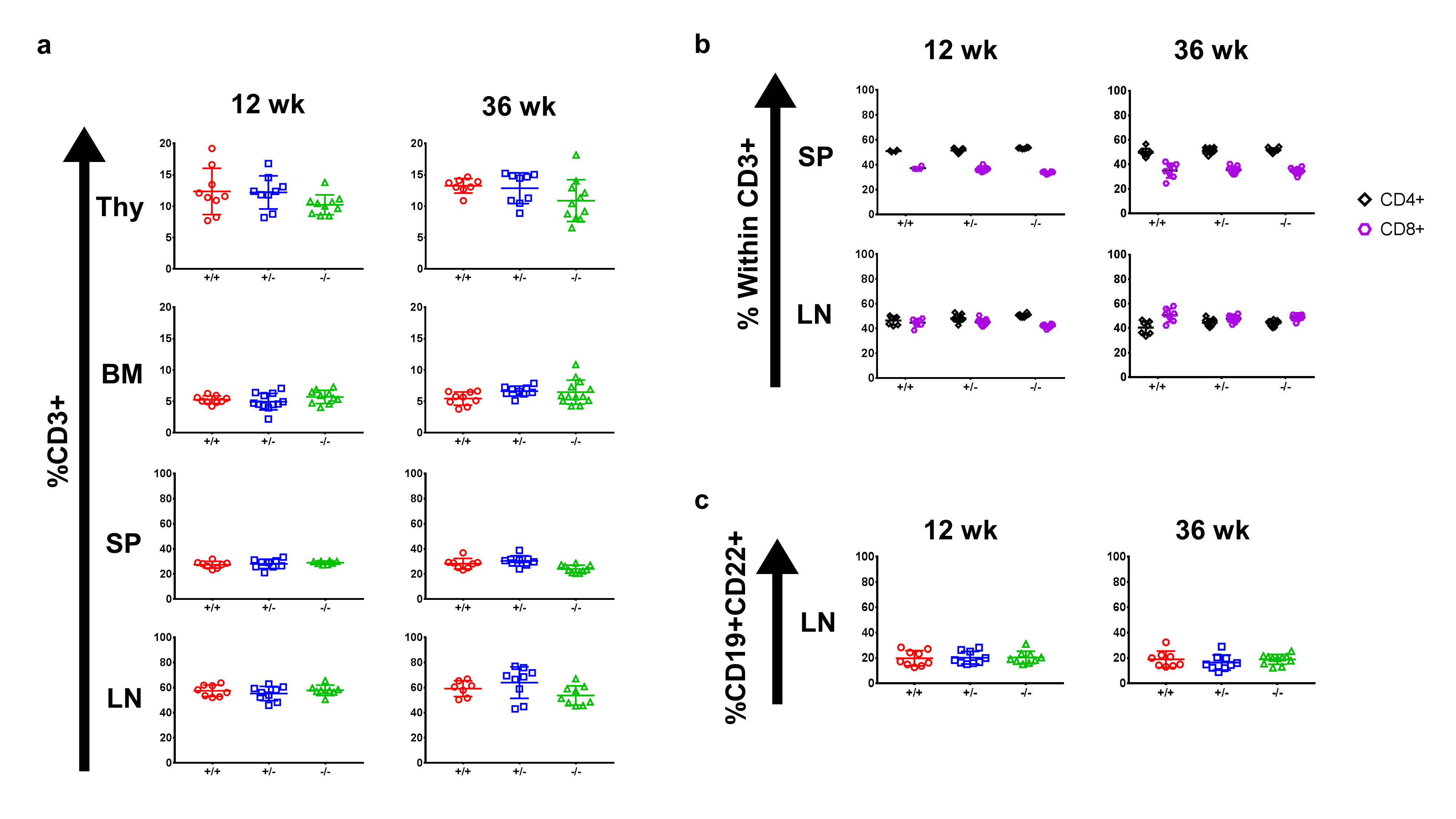

Supplement: Supplementary file 3 — Additional file 3: Figure S3. (a) Dot plots of CD3+ T cells in the Thy, BM, SP, and LN for listed genotypes at 12 and 36 weeks as determined by flow cytometry (n = 7–13 per group). (b) Dot plots of CD3+CD4+ and CD3+CD8+ T cell populations in the secondary lymphoid organs of listed genotype at 12 and 36 weeks (n = 4–9 per group). (c) Dot plots of CD19+CD22+ B cells in the LN for listed genotypes at 12 and 36 weeks (n = 8–11 per group). (Dot plots show mean ± SD with each dot corresponding to a biological replicate). [file 13104_2018_3583_MOESM3_ESM.tif]

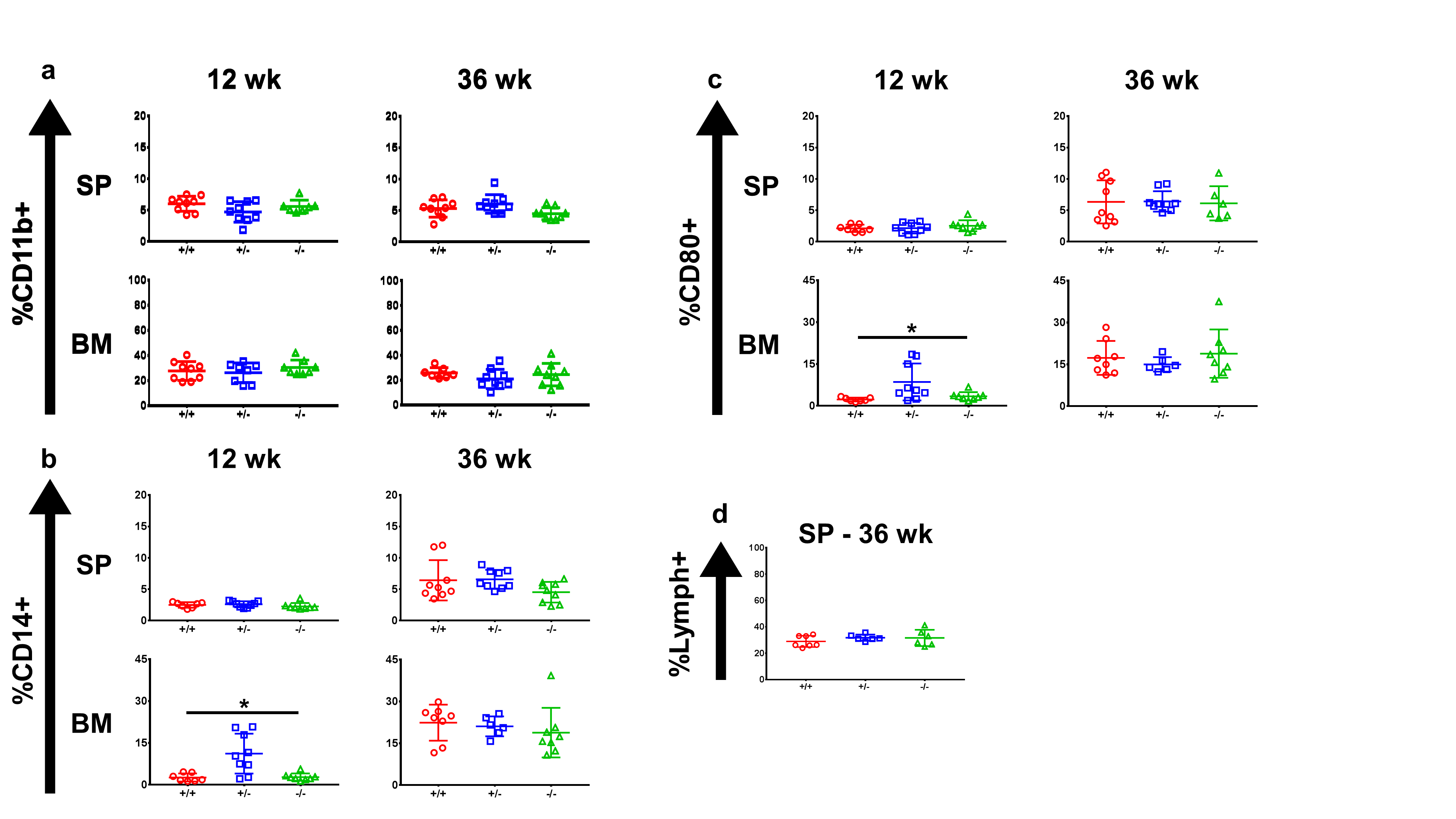

Supplement: Supplementary file 4 — Additional file 4: Figure S4. (a) Dot plots of CD11b+ cells in the SP and BM at the listed genotypes for 12 and 36 weeks. (b) Dot plots of CD14+ cells in the SP and BM at the listed genotypes for 12 and 36 weeks. (d) Dot plot of splenic lymphocyte percentage in 36-week mice for listed genotypes. (Dot plots show mean ± SD with each dot corresponding to a biological replicate, NS not significant, * = p < 0.05 ANOVA and Tukey post hoc test). [file 13104_2018_3583_MOESM4_ESM.tif]
