# Supplementary material for: Caveolin-1 is dispensable for early lymphoid development, but plays a role in the maintenance of the mature splenic microenvironment
Source: BMC Res Notes. 2018 Jul 13;11:470. doi: 10.1186/s13104-018-3583-3 (PMC6043983; doi:10.1186/s13104-018-3583-3)
Supplement: Supplementary file 5 — Additional file 5: Table S1. Histological scoring table. Table S2. Patient characteristics from the LY6161 high-density lymphoma tissue array. Table S3. Publicly available GEO DataSets utilized for gene expression analysis. Table S4. Antibodies utilized within the study. [file 13104_2018_3583_MOESM5_ESM.docx]

**Additional File 5**

**Additional file 5: Table S1.** Histological scoring table.

| **Histology scoring variable** | **Average measures intraclass correlation coefficient** | **95% Confidence interval** |
| --- | --- | --- |
| Ki67 staining | 0.9111 | 0.8401 to 0.9536 |
| Secondary follicle count | 0.9397 | 0.8677 to 0.9756 |
| Plasma cell count | 0.7970 | 0.6451 to 0.9652 |
| TMA scoring | 0.6053 | 0.5264 to 0.6730 |

**Additional file 5: Table S2.** Patient characteristics from the LY6161 high-density lymphoma tissue array.

| **Diagnosis** | **Sample #** | **Median Age (years)** | **Sex (%)** |
| --- | --- | --- | --- |
| Normal Lymph Node Tissue | 26 | 35 | M (78.6) F (21.4) |
| Follicular Lymphoma | 293 | 47 | M (55.6) F (44.4) |
| Diffuse B-cell Lymphoma | 18 | 49.5 | M (59.4) F (40.6) |

| **GEO Accessions** | **Sample Type & n-Value** | **PMIDs**^†^ |
| --- | --- | --- |
| GSE57944; GSE3526; GSE7307 | Normal Spleen (12) | 25057852; 16572319; N/A |
| GSE25638; GSE23501 | DLBCL-GCB^‡^ (40), DLBCL-ABC^§^ (46) | 22689981; 20610814 |
| GSE25638; GSE35426; GSE16024 | Follicular lymphoma (27) | 22689981; 23028731; N/A |

**Additional file 5: Table S3.** Publicly available GEO DataSets utilized for gene expression analysis.

^†^PubMed identifier

^‡^Diffuse large B-cell lymphoma germinal center B cell-like

^§^Diffuse large B-cell lymphoma activated B cell-like

**Additional file 5: Table S4.** Antibodies utilized within the study.

| **Flow Cytometry Antibodies**  (purchased from BD Biosciences) | | |
| --- | --- | --- |
| *Protein Antibody* | *Fluorophore* | *Clone* |
| **T-cell panel** | | |
| anit-CD3ε | PE-Cy7 | clone 145-2C11 |
| anti-CD4 | PE | clone GK1.5 |
| anti-CD8 | PerCP-Cy5.5 | clone 53-6.7 |
| **B-cell panel** | | |
| anti-CD19 | PE-Cy7 | clone 1D3 |
| anti-CD22.2 | FITC | clone Cy34.1 |
| anti-CD5 | PE | clone 53-7.3 |
| **Myeloid Panel** | | |
| anti-CD11b | PE-Cy7 | clone M1/70 |
| anti-CD14 | PE | clone rmC5-3 |
| anti-CD80 | PerCP-Cy5.5 | clone 16-10A1 |
| **Immunohistochemistry Antibody**  (purchased from Abcam) | | |
| Ki67 | IgG | clone SP6 |
