# Supplementary material for: Caveolin-1 is dispensable for early lymphoid development, but plays a role in the maintenance of the mature splenic microenvironment
Source: BMC Res Notes. 2018 Jul 13;11:470. doi: 10.1186/s13104-018-3583-3 (PMC6043983; doi:10.1186/s13104-018-3583-3)
Supplement: Supplementary file 6 — Additional file 6. Animal care and secondary follicle area determination methodologies are described. [file 13104_2018_3583_MOESM6_ESM.docx]

**Additional File 6**

**Additional Methods**

**Animal care**

C57BL/6J (stock: 000664) and B6.Cg-*Cav1^tm1Mls^*/J (stock: 007083) mice were purchased from the Jackson Laboratories and bred to generate *Cav1^+/-^* mice. All mice for experimental use were sacrificed +/- 1 week of age for the following time points: 12 weeks (“young/naïve”), 24 weeks, 36 weeks (“aged/mature”). Euthanasia was carried out using CO_2_ inhalation with the secondary method of cervical dislocation. All experimental protocols were reviewed and approved per the Institutional Animal Care and Use Committee at the University of Nebraska Medical Center/University of Nebraska at Omaha (IACUC# 13-056-08).

**Secondary follicle area determination**

An average of 9 (range: 5 – 10) representative secondary follicles were selected per mouse (n = 2 – 4 per genotype per timepoint) and traced using the “pen” tool for determination of total area (μm^2^).
